# Mr. Carlisle's Plan for a Convalescent Establishment

**Published:** 1801-04

**Authors:** Anthony Carlisle

**Affiliations:** Soho Square


### Medical and Physical Journal.

VOL. V.] APRIL, 1801. [NO. XXVI.

### *To the Editors of the Medical and Physical Journal.*

GENTLEMEN,

AS the public attention is just now attracted towards a Plan for the establishment of receiving Houses for sick and convalescent Poor in the Metropolis, it is but an act of justice due to the Public, as well as to the original Projectors of a similar plan, that it should be also made known, in order that powerfully benevolent men should avail themselves of whatever good may be contained in the unpublished scheme alluded to.

In the spring of 1798, the following project for instituting a Convalescent Asylum, under the auspices of the Governors of the three Hospitals of Westminster, St. George's, and the Middlesex, was laid before a few of my particular friends,* by whose co-operation many of the details for conducting the Institution were drawn up, and the whole affair seemed ripe for promulgation, when the consideration of the peculiar situation of the country, and the financial limitations imposed even upon the opulent, deterred us from making any further attempt. In addition to this, another very powerful obstacle arose, which effectually checked me, as the original projector, in my future proceedings; and this was, the sure prospect of all that train of opposition, jealousy, and calumny which seem to be unavoidably the lot of those who are likely to gain honor and fame by even doing good. Your publication, as a vehicle for whatever temporary matter influences the profession or science of medicine, may probably not prove an improper channel for communicating the Outlines of this Scheme, more especially as (if worth attention) it will apply to the Hospitals in other parts of the metropolis, as well as to those in all large towns.

* Doctor Bradley; Mr. Saxon, architect; G. C. Bedford, Esq. John May, Esq. and Mr. Robert Southey.

The confiderations which led to the notion of projecting a plan for eftablifhing an Afylum for the Convalefcents of the great public Hofpitals in London, were derived from facts which had fallen under my own obfervation. I had often traced the paths of individual paupers, difcharged from Hofpitals in a convalefcent ftate, (for it may be obferved that few patients leave an hofpital in perfect health) and had generally found them finking under the accumulated impediments of immediate poverty, lofs of their former occupation, and want of all the means to fupport and comfort a debilitated frame. From thefe caufes they either returned to the Hofpital, or, defpairing of effectual relief, funk under their diftreffes, or were thrown upon the parifh work-houfe. Many fuch cafes occurred to my own knowledge; and it feemed highly probable that the Subfcribers to Hofpitals for relieving fick and lame poor, would gladly fupport any inftitution for the more effectually completing their benevolent wifhes. The numerous wants which attend a poor man difcharged in a ftate of returning health from a Hofpital, prefented themfelves as almoft infurmountable difficulties; moft of thefe, however, were in theory overcome. To return to his empty garret badly cloathed, pennylefs, and uncertain of that employment which his mean ftate hardly enables him to perform, is a profpect which cannot be borne with cheerfulnefs; and hence the almoft univerfal defire among the poor to remain in a public Hofpital after they are cured of their bodily complaints. In thofe cafes where a family depends on the labour of a parent, the fcene is ftill more diftreffing; and where children are returned half fick to their parents, they feldom meet with the needful means of recovery.

The bad air of the metropolis alone feemed a fufficient objection againft convalefcent eftablifhments within the town; neither could any fort of healthy exercife be expected from fimple manufacturing labour to be performed within doors. The idea of a public kitchen and fruit garden, fituated within a mile or two of London, embraced every defirable quality; and as a confiderable profit would accrue from the fale of the produce of an extenfive garden of this defcription, it appeared above all others the moft eligible for our purpofe. The plan then grew up, and the neceffary enquiries were made. The number of acres to be cultivated by the number of convalefcents, with the feveral occupations of each defcription of the lame or infirm, were laid down; the expences of building and enclofing, the plan of which was admirably drawn out by Mr. Saxon; the total expence of mafter and miftrefs, fervants, two horfes, market waggon, &c.; rent of land, diet of convalefcents, were eftimated to leave a fair recompence in money for each each convalefcent when difcharged from this afylum. The particulars of this project were given by Mr. May to the Society for bettering the Condition of the Poor, about the time of its firft origin; and I think it was delivered by myfelf to Count Rumford, who approved of feveral parts, propofed fome amendments, and queries. Here the bufinefs has refted; but if any of your Readers wifh for the whole particulars, drawings, &c. I will gladly deliver them over to him, together with any other information I may poffefs on this fubject.

*Soho Square,* I am, your's, &c.

*March* 21, 1801. ANTHONY CARLISLE.

